# Insulin expressing hepatocytes not destroyed in transgenic NOD mice

**DOI:** 10.1186/1740-2557-1-3

**Published:** 2004-11-08

**Authors:** Muhammad T Tabiin, Christopher P White, Grant Morahan, Bernard E Tuch

**Affiliations:** 1 Diabetes Transplant Unit, Prince of Wales Hospital, The University of New South Wales, Sydney, Australia; 2 Joslin Diabetes Centre, Harvard Medical School, Boston, Massachusetts, USA; 3 Walter Eliza Hall Institute of Medical Research, Melbourne, Australia

## Abstract

**Background:**

The liver has been suggested as a suitable target organ for gene therapy of Type 1 diabetes. However, the fundamental issue whether insulin-secreting hepatocytes *in vivo *will be destroyed by the autoimmune processes that kill pancreatic β cells has not been fully addressed. It is possible that the insulin secreting liver cells will be destroyed by the immune system because hepatocytes express major histocompatibility complex (MHC) class I molecules and exhibit constitutive Fas expression; moreover the liver has antigen presenting activity. Together with previous reports that proinsulin is a possible autoantigen in the development of Type 1 diabetes, the autoimmune destruction of insulin producing liver cells is a distinct possibility.

**Methods:**

To address this question, transgenic Non-Obese Diabetic (NOD) mice which express insulin in the liver were made using the Phosphoenolpyruvate Carboxykinase (PEPCK) promoter to drive the mouse insulin I gene (Ins).

**Results:**

The liver cells were found to possess preproinsulin mRNA, translate (pro)insulin *in vivo *and release it when exposed to 100 nmol/l glucagon *in vitro*. The amount of insulin produced was however significantly lower than that produced by the pancreas. The transgenic PEPCK-Ins NOD mice became diabetic at 20–25 weeks of age, with blood glucose levels of 24.1 ± 1.7 mmol/l. Haematoxylin and eosin staining of liver sections from these transgenic NOD PEPCK-Ins mice revealed the absence of an infiltrate of immune cells, a feature that characterised the pancreatic islets of these mice.

**Conclusions:**

These data show that hepatocytes induced to produce (pro)insulin in NOD mice are not destroyed by an ongoing autoimmune response; furthermore the expression of (pro)insulin in hepatocytes is insufficient to prevent development of diabetes in NOD mice. These results support the use of liver cells as a potential therapy for type 1 diabetes. However it is possible that a certain threshold level of (pro)insulin production might have to be reached to trigger the autoimmune response.

## Background

Genetic alteration of non-pancreatic cells in a diabetic person to synthesise, store and secrete insulin in the same manner as a pancreatic β cell is a potential therapy of type 1 diabetes. The hepatocyte has been suggested as a suitable target cell for such gene therapy [1-9]. Such cells made *in vitro *are capable of synthesizing and storing pro(insulin) and can secrete this peptide in response to a physiological challenge with glucose [1,2]. Moreover, when transplanted into diabetic mice these cells can lower blood glucose levels to the normal range [2]. Further, Ferber et al. [9] has recently showed that adenovirus-mediated *in vivo *transfer of the PDX-1 transgene to the mouse liver results in the conversion of a hepatocyte subpopulation to the beta cell phenotype. Ferber also showed that this population of trans-differentiated liver cells was induced to produce the prohormone convertases PC1/3 and PC2 leading to complete processing of proinsulin. The amount of insulin produced was sufficient to ameliorate streptozotocin-induced hyperglycaemia in the mice.

For this gene therapy approach to be viable, the insulin producing liver cells must not be destroyed by the immune system or else this could lead to liver damage. This fundamental question has not been addressed by previous studies with (pro)insulin producing hepatocytes as diabetes in these models was induced chemically rather than by autoimmune means [6-9]. The autoimmune destruction of insulin-producing hepatocytes is a possibility since hepatocytes express major histocompatibility complex (MHC) class I molecules [10], and constitutively express Fas [11], moreover the liver cells have antigen presenting activity [12] and are attacked in autoimmune diseases such as chronic active hepatitis. Furthermore (pro)insulin appears to be an autoantigen in the development of type 1 diabetes [13,14]. Evidence for this comes from the presence insulin and Glutamic Acid Decarboxylase (GAD) autoantibodies in the sera of people with prediabetes [13]. It also comes from studies that show that diabetes can be adoptively transferred to normoglycaemic mice by the introduction of insulin-specific T cell clones [14]. These data taken together suggest that hepatocytes which produce (pro)insulin might be targeted and destroyed by the same autoimmune processes responsible for the destruction of pancreatic β cells.

To address this question *in vivo*, transgenic Non-Obese Diabetic (NOD) mice that express insulin in the liver were created using the Phosphoenolpyruvate Carboxykinase (PEPCK) promoter [15] to direct expression of the mouse insulin I gene. This promoter had previously been used to create a transgenic C57BL/6 human insulin secreting mouse line with hepatic insulin expression [7]. However these mice do not develop autoimmune diabetes and would be unsatisfactory to address the issue of insulin autoantigenicity.

The liver of the transgenic PEPCK-Ins NOD mice were characterised with respect to insulin mRNA transcription, (pro)insulin content and (pro)insulin release. The blood sugar levels of the animals were monitored and the livers of the animals were analysed for any evidence of immune cell infiltration.

## Methods

### Materials

The PEPCK promoter was generously donated by Dr R. W. Hanson (Case Western Reserve University, Cleveland, Ohio, USA). DNA modifying enzymes and competent bacteria for transformation were purchased from Promega (Madison, Wisconsin, USA). RPMI 1640, α-MEM and fetal bovine serum (FBS) were purchased from Trace Biosciences (Castle Hill, Sydney, Australia). Hybond-N+ nylon membrane for the Southern Blot and ribonuclease protection assay (RPA) was from Amersham International (Bucks, UK). Human insulin and rat insulin standards for the in-house radioimmunoassay (RIA) were obtained from Novo Nordisk (Bagsvaerd, Denmark).

### Transient transfection of HEPG2 cells

The transfection of the HEP G2 cells with the PEPCK-Ins transgene was carried out using Lipofectamine (GibcoBRL, Madison, Wisconsin, USA). Cells were trypsin harvested and seeded 24 hr prior to transfection at a density of 200,000 cells/well in 24 well tissue culture plates (Becton Dickinson, New Jersey, USA) to achieve 60–80% confluence at the time of transfection. 10μl of Lipofectamine was added to 300μl of serum free media and incubated at room temperature for 30 minutes. After the incubation, 300μl of DNA solution (3–4μg) was added and the lipid:DNA complex solution incubated at room temperature for 15 minutes. The cells were washed once with 1 ml of serum free medium prior to the transfer of 300μl of the lipid:DNA complex solutions to the cells. The cells were then incubated for 5 h at 37°C in an air incubator. The DNA/lipid complex media was removed and replaced with 2 mL of α-MEM 10% FBS which contains Antimycotic and Antibiotic solution (10μL/mL) (Sigma, St Louis, Missouri, USA). In addition, for the PEPCK-Ins transgene, a final concentration of 100 nM glucagon was added to the media to upregulate the PEPCK promoter. The cells were incubated at 37°C in an air incubator for 48–60 hr. After the incubation, the conditioned culture medium was collected and assayed for mouse (pro)insulin.

### Insulin Radioimmunoassay (RIA)

(Pro)insulin was measured by an in-house RIA using rat insulin standard (Novo Nordisk Laboratories, Bagsvaerd, Denmark). The lowest value on the rat insulin standard curve used in the assay was 0.25 ng/ml. The intra and interassay coefficients of variance were <5% and 10% respectively.

### Generation of transgenic mice

**Figure 1:**
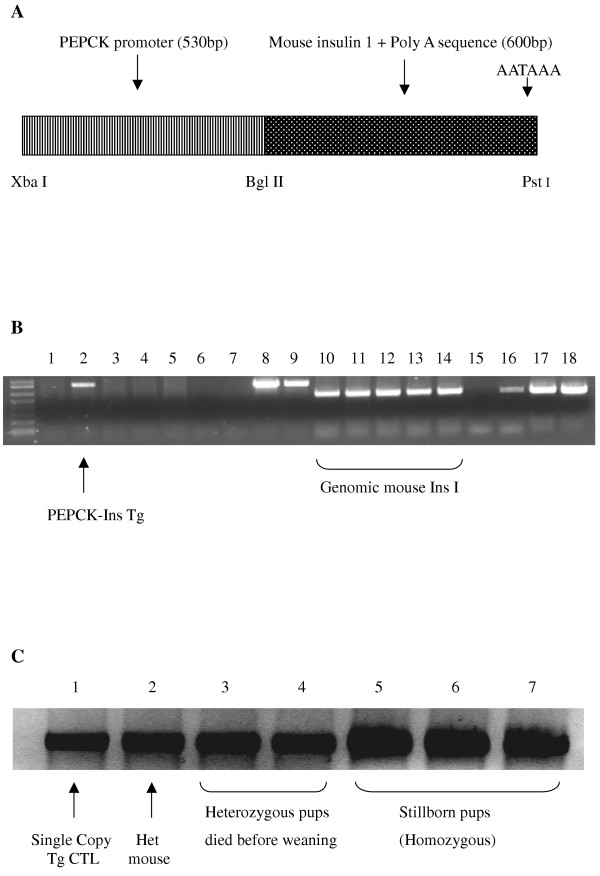
**A-C**. The PEPCK-Ins transgene. Schematic representation (**A**). Example of PCR screening of PEPCK-Ins transgenic mice (**B**). Lanes 1–5 and lanes 10–14 are PCR reactions with genomic DNA extracted from mice that developed from microinjected mouse eggs. Primers specific for the PEPCK-Ins transgene were used for lanes 1–9 (30 cycles) while primers specific for mouse insulin 1 were used for lanes 10–18 (30 cycles). Lanes 6 and 15 are PCR negative controls (no DNA added). Lanes 7 and 16 are PCR reactions to demonstrate the specificity of the primers used (wild type NOD mouse DNA added). Lanes 8, 9, 17 and 18 are half copy spiked and plasmid controls respectively (positive controls). Example of Southern Blotting for the PEPCK-Ins transgene (**C**). Lane 1 One copy spiked sample (100 ng Balb/c genomic DNA + 102fg PEPCK-Ins plasmid), lane 2 genomic DNA from tail tip of F2 PEPCK-Ins mouse, lanes 3 and 4 are DNA from pups which died before weaning and lanes 5–7 are DNA from still born pups. Each lane was loaded with 15μg of genomic DNA that was digested with Xba I and Pst I to release the transgene.

To direct the expression of the mouse insulin I gene to the liver of the transgenic mice, the 5' flanking sequences (-460 bp to +73 bp) of the rat PEPCK gene [15] were used to drive the expression of the genomic mouse insulin I gene (-25 bp to +557 bp) [16]. The construct was digested with Xba I and Pst I (Fig 1A) and the PEPCK-Ins transgene was isolated from the agarose gel using the Supelco GenElute Spin Column (Sigma, St Louis, Missouri, USA). Standard procedures were followed to generate transgenic mice [17]. Fertilized mouse eggs were flushed from the oviducts of superovulated NOD mice 6–8 h after ovulation. Male pronuclei of the fertilized eggs were injected with 2pl of the DNA solution (2 ng/μl) and the embryos that divide to the 2 cell stage were implanted in the oviducts of pseudopregnant mice. After birth the animals were tested for the presence of the transgene by PCR and Southern blot of the DNA from tail tip samples taken after weaning at 3 weeks of age.

### Treatment of the animals

The mice were fed ad libitum with a standard diet and kept under a light-dark cycle of 12 h in compliance with the animal ethics of our institution. The facilities used to breed and maintain the mice were specific pathogen free, with air passaged through a HEPA filter. The transgenic PEPCK-Ins NOD mice and the wild type littermates were monitored for natural development of diabetes. The diabetic PEPCK-Ins transgenic NOD mice were maintained by daily insulin injections and sacrificed 4 weeks after they became diabetic.

### Blood glucose measurements of mice

Blood glucose levels of the wild type as well as the transgenic NOD mice were performed using the Medisense Precision QID Blood Glucose Sensor System (Bedford, Massachusetts, USA). The mice were bled by pricking the tail vein and 5μl of the blood placed on to the Precision Plus blood glucose electrode. A mouse was classified as being diabetic if a reading of ≥ 15 mmol/l was obtained on more than one occasion.

### Insulin mRNA analysis

The total RNA from liver and pancreas was isolated using TRIzol (Life Technologies, Grand Island, New York, USA) [18]. The detection of mouse insulin I mRNA by RT-PCR was performed using Superscript II RNase H^- ^Reverse Transcriptase from GibcoBRL (Grand Island, New York, USA) to synthesize the first strand cDNA as recommended by the manufacturer. The cDNA sample was then used as a template in PCR amplification (30 cycles) using primers specific for mouse insulin I mRNA. The sequence of primers used were:

(Forward) TAA CCC CCA GCC CTT AGT GAC CAG CTA TAA

(Reverse) AAA GTT TTA TTC ATT GCA GAG GGG TGG GGC

The PCR products were run on a 1.2% Tris Acetate EDTA agarose gel, photographed and analysed using the Gel Doc 1000 system (Biorad, Hercules, California, USA).

Insulin mRNA in the samples was also visualised using the ribonuclease protection assay (RPA) method. The plasmid used as the template to transcribe the riboprobe for mouse insulin I was a generous gift from Chirgwin J. M. (San Antonio, Texas, USA). The Digoxigenin (DIG) labelled insulin riboprobe was synthesized by the *in vitro *transcription method using the DIG RNA labeling kit (SP6/T7) from Boehringer Mannheim (Mannheim, Germany), which utilises DIG-dUTP, according to the manufacturer's recommendations.

Hybridisation and RNase treatment were performed using the RPA II kit from Ambion according to the manufacturer's recommendations. Next the samples were loaded and run on a 6% polyacrylamide/8 M urea denaturing mini-gel in Tris borate EDTA buffer. The RNA bands on the gel were transferred onto a nylon membrane by using an electro-gel transfer apparatus and the membrane was fixed by using an UV cross linker (Hybaid, UK).

For the visualisation of the RNA bands on the nylon membrane, the DIG Wash and Block Buffer Set and the DIG Chemiluminescent Detection Kit, both of which were purchased from Boehringer Mannheim, were used. The membrane was then exposed to a standard Kodak X-ray film for 30 minutes. The film was subsequently analysed using the Gel Doc 1000 system.

### Organ culture

Pancreatic and hepatic tissue organ cultures were performed using organ culture dishes from Becton Dickinson (New Jersey, USA) [19]. Briefly, the organs were taken from mice, and an aliquot of 10–30 mg removed from each organ. The tissue was then diced into 1 mm^3 ^explants and spread on a filter paper placed on a sterile wire grid in the inner well of the organ culture dish such that the tissue was exposed to air above and the RPMI 1640 10% FBS medium below. Sterile saline was placed in the outer well to maintain a humidified environment. The tissue in the organ culture dishes were incubated for 24 hr in a humidified 5% CO_2 _and air incubator at 37°C. Culture supernatant from all media changes of respective wells were pooled separately and kept at -20°C until the RIA was performed. The results were expressed per mg of tissue.

### (Pro)insulin content

(Pro)insulin was extracted by homogenising a weighed sample of liver or pancreas in acid-ethanol (solution of 0.18 mol/l HCl in 70% ethanol) and incubating overnight at 4°C. The next day, the samples were spun down and the supernatant removed. The (pro)insulin content was determined by RIA and the results expressed per mg tissue.

### In situ hybridisation of (pro)insulin mRNA

To detect the transcription expression of (pro)insulin, in situ hybridisation (ISH) was performed on pancreatic and liver tissues of diabetic transgenic PEPCK-Ins NOD mice and diabetic wild type NOD mice using a modification of a previously described method (20). Briefly, 4μm thick paraffin sections were deparaffinized with xylene, ethanol and air dried. The sections in 0.01 mol/l citric buffer, pH 6.0 were then treated with microwave irradiation for 10 min. Following this procedure the sections were treated with proteinase K (Invitrogen, Carlsbad, CA, USA) 1μg/ml for 15 min at 37°C. They were washed 3 times with phosphate buffered saline for 5 min and dried with ethanol. The sections were then hybridized with [35]-S-labelled riboprobes (1μg/ml) overnight at 57°C. The [35]-S-labelled riboprobes were synthesized by in vitro transcription (SP6/T7) incorporation of [35]-S-dUTP using RNA polymerase (Roche, Mannheim, Germany). The template used was mouse insulin I cDNA that was generously donated by Chirgwin JM (San Antonio, Texas, USA). The hybridisation buffer consisted of a 25μl hybridisation cocktail (labelled riboprobe 500,000 cpm/section, 50% formamide, 0.1% SDS, 0.1% sodium thiosulfate, 0.1 mol/l DTT, 0.3 mol/l NaCl, 20 mmol/l Tris-HCl [pH 7.5], 2 mmol/l EDTA, 20% dextran sulphate, 0.02% sheared salmon sperm DNA, 0.1% total yeast RNA, 0.02% yeast tRNA, 1 × Denhardt's solution). After hybridisation, the slides were soaked in 2X standard saline citrate (SSC), rinsed with deionised water and then treated with RNase A (20μg/ml) for 30 min at room temperature. High stringency washes were then performed on the slides with 2XSSC followed by 0.2XSSC. The slides were then dried by ethanol, air dried for 20 min and the results viewed by NBT2 emulsion autoradiography (Kodak, Rochester, New York). For controls, sense-probe instead of anti-sense was used for hybridisation. The photos were taken using the Olympus IX70 microscope (Melville, New York) with a dark field condenser.

### Histochemistry

Sections of the liver and pancreas from the transgenic mice were washed twice in Phosphate Buffered Saline (PBS), fixed in 10% formalin overnight and embedded into paraffin. Consecutive 5μm sections were cut and placed on poly-L-lysine-coated slides. After dewaxing and serial alcohol rehydration, the tissue sections were treated with H_2_O_2_, and then incubated in PBS containing 10% goat serum or 1% BSA for 20 minutes at room temperature to block nonspecific binding.

For insulin staining, primary insulin antibody (Dako Corporation, Via Real, Carpinteria, California, USA) at 1:1250 was added and the sections were incubated overnight. The next morning the sections were washed in PBS and incubated with anti-guinea pig IgG (1:400) at room temperature for 20 minutes. Thereafter the sections were washed again with PBS, and biotinylated anti-rabbit / mouse antibody added to the sections for 15 minutes followed by streptavidin-peroxidase conjugate for a further 15 minutes. Finally, the sections were treated with substrate-chromogen AEC. A standard concentration (0.1%) of haematoxylin was added as a counterstain. The primary antibody was omitted for the negative control. The haematoxylin and eosin staining of sections was performed automatically by the Jung Autostainer XL machine (Leica, New Jersey, USA).

### Statistical analysis

The log rank-test was used to determine whether the occurrence of diabetes in the transgenic mice was significantly different from the wild type NOD mice. The non-parametric t-test (Mann-Whitney U test) was used to determine whether the blood glucose levels of the diabetic transgenic and wild type NOD mice were significantly different.

## Results

### Transient transfection of HEP G2 with PEPCK-Ins

To confirm that the PEPCK-Ins transgene (Figure 1A) was functional, 3–4μg of the transgene was transfected into the human hepatoma cell line HEP G2. After the transfection, the cells were incubated in α-MEM supplemented with 10% FBS. Glucagon was added to upregulate the PEPCK promoter expression at a final concentration of 100 nmol/l for 24 hr. At the end of the incubation period, the conditioned culture medium was positive for rodent insulin at a concentration of 0.90 ± 0.04 ng/200,000 HEP G2 cells (n = 8).

### Generation of transgenic mice

From a series of two microinjections, 43 mice were obtained of which 4 were positive for the PEPCK-Ins transgene when analysed by PCR (Figure 1B) and Southern blot (Figure 1C). Two of the 4 transgenic founder mice were found to have established germline transmission of the transgene. From the Southern blot analysis, these mice have only a single copy of the transgene (Figure 1C).

### Transcription of insulin I mRNA in the liver

**Figure 2:**
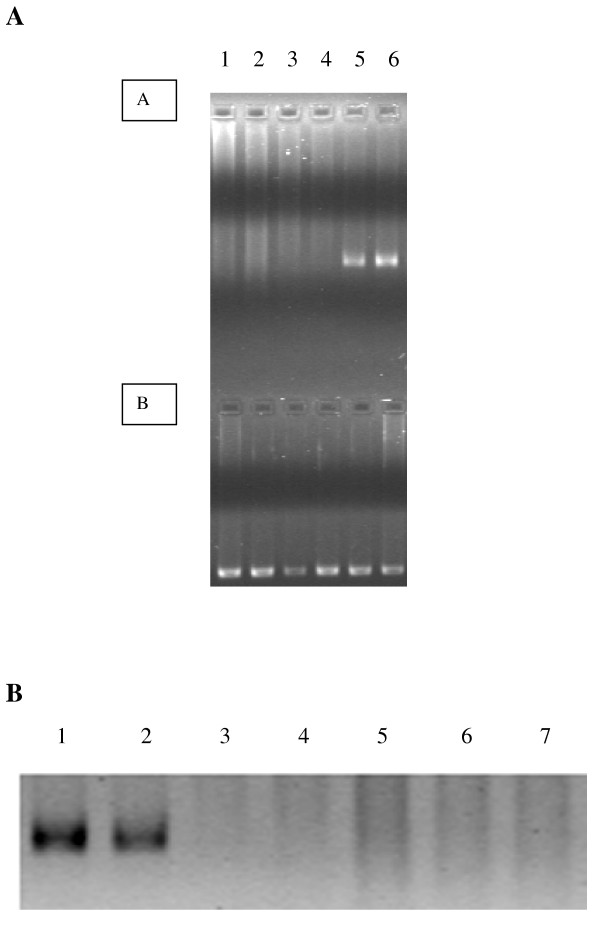
**A-B**. Mouse insulin 1 mRNA. RT-PCR (**A**). Lanes 1A-6A are RT-PCR reactions using primers specific for mouse insulin 1 mRNA (30 cycles). Lanes 1B-6B are RT-PCR reactions using primers specific for mouse GAPDH mRNA (30 cycles). Lanes 1–4 are total RNA from the liver of progeny from founder T645-18, lane 5 is total RNA from the liver of progeny from founder T647-2 and lane 6 total RNA from the pancreas of progeny from founder T645-18. RPA for mouse insulin 1 mRNA (**B**). Lane 1 pancreatic total RNA, lane 2 liver total RNA from T647-2 progeny and lanes 3–7 liver total RNA from T645-18 progeny.

Of the two lines only one was found to transcribe insulin mRNA in the liver, as determined by mouse insulin I RT-PCR (Figure 2A) and mouse insulin I Ribonuclease Protection Assay (RPA) (Figure 2B). No insulin mRNA was detected in the second line despite documented germline transmission of the transgene. The transgenic mice appeared healthy and active, and were normoglycaemic. The blood glucose levels were 6.0 ± 1.1 mmol/l (n = 20) as compared to 6.5 ± 0.5 (n = 20) for wild type littermates.

### Production of (pro)insulin from liver cells

The ability of the liver cells from PEPCK-Ins positive transgenic mice to produce (pro)insulin was examined on liver removed from mice that had been sacrificed. Ideally production of (pro)insulin from the liver would be sought *in vivo*. This was not possible since any (pro)insulin released from the liver would be indistinguishable from that secreted from pancreatic β cells. Two methods were used to analyse if (pro)insulin was produced from liver tissue removed from the mice. The liver was both extracted for its hormonal content, and placed in organ culture with analysis of the conditioned culture medium for (pro)insulin. In both cases, (pro)insulin was measurable. The (pro)insulin content was 0.5 ± 1 ng/mg (n = 5), which is equivalent to 8% of the (pro)insulin content of the pancreas (Table 1) on a weight to weight basis. Organ culture of explants in the presence of 100 nM glucagon resulted in release of 3.4 ± 1 ng/mg (pro)insulin per day, which is 23% of that produced by cultured pancreatic explants. No (pro)insulin could be found either in liver extracts or from cultured liver explants taken from wild type NOD mice. These results show that there was translation of (pro)insulin from preproinsulin mRNA in the liver of transgenic mice. The amount of (pro)insulin present in the liver cells was too small for it to be detected immunohistochemically (data not shown).

**Table 1:** (Pro)insulin content of and release from pancreatic and hepatic tissue of transgenic and wild type NOD mice.

	**PANCREAS**		**LIVER**	
F2 progeny	(Pro)insulin content (ng/mg)	(Pro)insulin secretion (ng/mg tissue/ day)	(Pro)insulin content (ng/mg)	(Pro)insulin release in the presence of 100 nmol/l glucagon (ng/mg tissue/ day)
Normoglycaemic transgenic (n = 5)	6.3 ± 0.8	15.8 ± 1.6	0.5 ± 0.1	3.4 ± 1.0
Normoglycaemic wild type (n = 3)	6.3 ± 1.0	15.2 ± 2.3	ND	ND
Diabetic transgenic (n = 5)	0.5 ± 0.1 (n = 3; 2 ND)	ND (n = 3)	0.6 ± 0.2 (n = 5)	3.6 ± 0.3 (n = 3)

ND – not detectable (<0.025 ng/mg tissue)

Data expressed as X ± SD.

### In situ hybridisation of (pro)insulin mRNA

**Figure 3:**
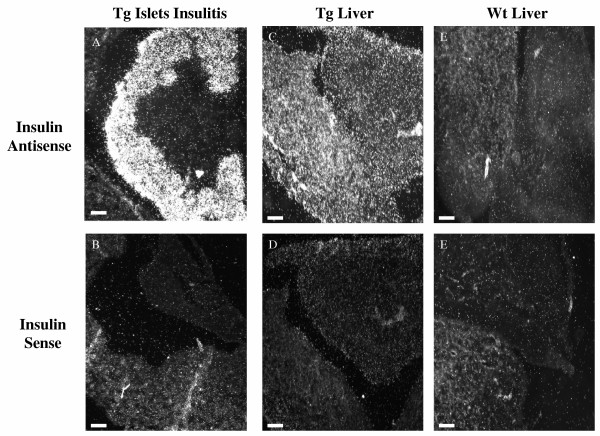
**A-F**. In-situ hybridisation. Pancreatic islets from a diabetic transgenic mouse illustrating insulitis, (**A**) antisense and (**B**) sense. Transgenic liver from a diabetic transgenic mouse (**C**) antisense and (**D**) sense. Wild type liver from a diabetic NOD mouse (**E**) antisense and (**F**) sense.

In situ hybridisation confirmed the presence of (pro)insulin mRNA in the pancreatic and liver sections of diabetic PEPCK-Ins transgenic mice (Figure 3A and 3C) but not in the liver section of the wild type NOD mice (Figure 3E). The (pro)insulin mRNA was localised to the hepatocytes of the diabetic PEPCK-Ins transgenic mice which appear to be evenly distributed throughout the liver section (Figure 3C). The sense control sections were negative (Figure 3B,3D and 3E).

### Occurrence of diabetes in transgenic mice

**Figure 4:**
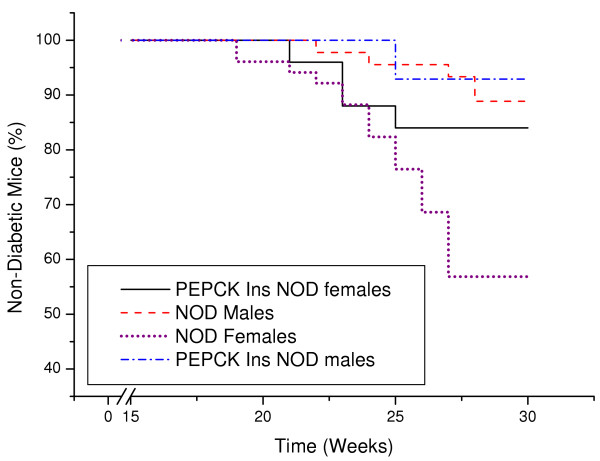
Incidence of diabetes in F1 and F2 PEPCK-Ins transgenic NOD mice versus wild type F1 and F2 NOD mice. PEPCK-Ins NOD males total n = 14, PEPCK-Ins NOD females n = 25, wild type NOD males n = 45 and wild type NOD females n = 51.

One out of 14 males (7%) and four out of 25 females (16%) of the F1 and F2 progeny of the PEPCK-Ins transgenic NOD mice became diabetic (Figure 4). The age at which this occurred was 20–25 weeks. Among the wild type NOD mice in our colony, 5 males out of 45 (11%) and 22 out of 51 females (43%) became diabetic (Figure 4), at 19–28 weeks of age. The mice were followed for a total of 15 months but no other transgenic mice became diabetic while 55% of the NOD females became diabetic. The incidence of diabetes in the transgenic PEPCK-Ins NOD females (16% diabetic at 30 weeks) differs significantly from the incidence of diabetes in the wild-type NOD females (43% diabetic at 30 weeks) as determined by the log-rank test (chi squared value 16.1, P < 0.001). The overall incidence of diabetes in the transgenic PEPCK-Ins NOD mice (13%) was also significantly lower compared to the wild type (28%) NOD mice as determined by the log-rank test (chi squared value 18.7, P < 0.001). The blood glucose levels of the diabetic transgenic NOD PEPCK-Ins mice (24.8 ± 1.9 mmol/l) were also significantly lower than those of the diabetic wild type NOD mice (>33 ± 2.1 mmol/l) as determined by the Mann Whitney U test, U = 32 and P = 0.004.

**Figure 5:**
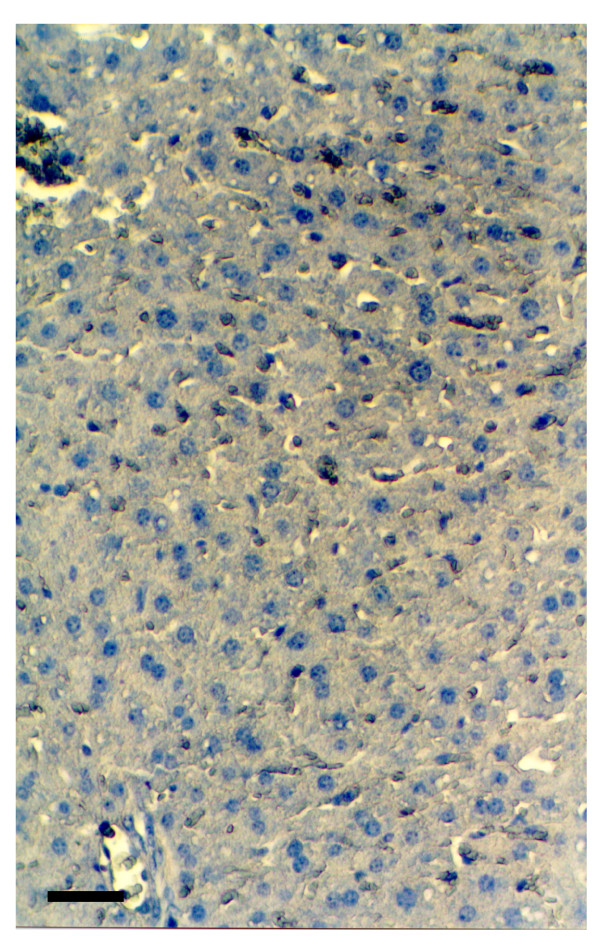
Staining of the liver of diabetic transgenic PEPCK-Ins mice with haematoxylin and eosin. (Black Bar = 20μm).

**Figure 6:**
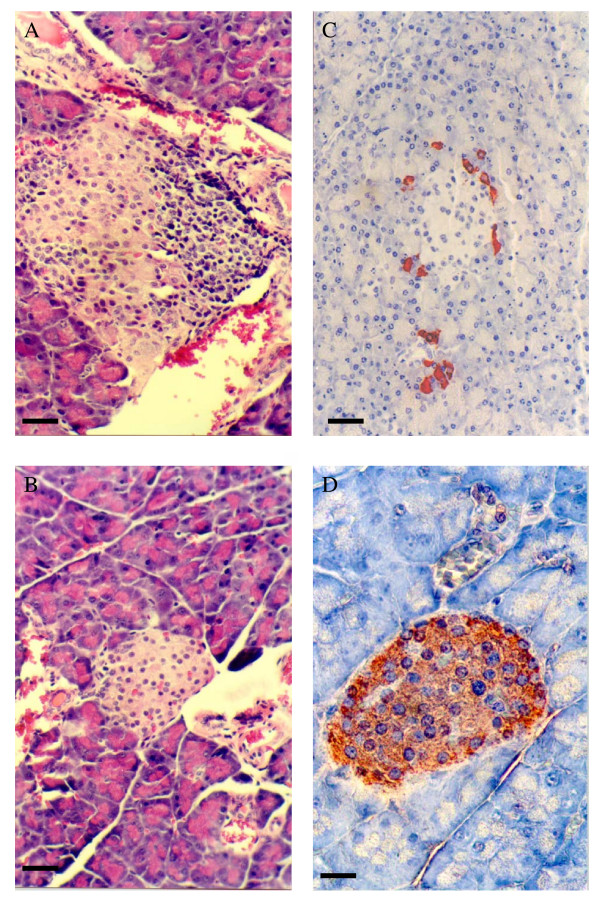
**A-D**. Haematoxylin and eosin, and insulin staining of pancreatic sections from transgenic PEPCK-Ins NOD mice. H & E staining of a pancreatic islet from a diabetic transgenic mouse illustrating insulitis (**A**), and from a normoglycaemic transgenic mouse (**B**). Insulin staining of a pancreatic islet from a diabetic transgenic mouse showing few remaining β cells (**C**) and from a normoglycaemic transgenic mice (**D**). Black Bar = 20μm for A-C, and 40μm for D.

The livers of the diabetic transgenic mice were examined histologically to determine if there was cellular infiltration and destruction of hepatocytes. This was not so, the liver having a normal architecture with no evidence of necrosis (Figure 5). In contrast, there was a cellular infiltrate in the islets of the diabetic (Figure 6A) but not normoglycaemic transgenic mice (Figure 6B) Immunohistochemical staining for (pro)insulin showed a reduced number of β cells (Figure 6C) compared to normoglycaemic littermates (Figure 6D).

Next, the (pro)insulin content of the liver of the diabetic mice was determined. This was similar to that from the liver of normoglycaemic transgenic mice (Table 1). (Pro)insulin release from cultures of liver cells was measured, and again found to be similar to that from the liver cells of non-diabetic transgenic mice (Table 1). However, as expected, the (pro)insulin content of the pancreas of diabetic transgenic mice was lower than that of normoglycaemic transgenic or wild type mice (Table 1). Indeed, the level was so low as to be comparable to that seen in the liver of either normoglycaemic or diabetic transgenic mice. The amount of (pro)insulin released from pancreatic explants from the diabetic transgenic mice was too low to be detected.

## Discussion

At the end of the study, 5 heterozygous transgenic PEPCK-Ins NOD mice had become diabetic. Nevertheless, preproinsulin mRNA was localised to the hepatocytes by in situ hybridisation and was detected in total RNA extracts of the livers of these transgenic mice by both RT-PCR and RPA. (Pro)insulin was detected in acid ethanol extracts of the livers from the transgenic mice by RIA. Furthermore, (pro)insulin was released from explants of transgenic liver placed in organ culture. These results indicate that the liver cells from these transgenic PEPCK-Ins NOD mice did synthesise and produce (pro)insulin.

Histological examination of the livers of the diabetic transgenic mice showed no infiltration of the liver by immune cells even though insulitis was observed in the pancreatic sections. This indicates that hepatic insulin production did not cause the development of tolerance to insulin in these PEPCK-Ins transgenic NOD mice. The lack of infiltration of immune cells in the livers of the diabetic transgenic mice also suggests that the mouse insulin I mRNA transcribing hepatocytes were not targeted or destroyed by the immune system. In addition, as the transgenic mice were sacrificed 4 weeks after the onset of diabetes, this reduces the possibility of delayed destruction of the (pro)insulin expressing hepatocytes. During this time, hepatic insulin expression was at least equal to that found in the pancreas. At the apparently low levels of transgene expression, these results suggest that (pro)insulin is not targeted by the immune system in this transgenic PEPCK-Ins mouse model superimposed on the autoimmune diabetes model of the NOD mouse. Likewise, Lipes et al reported that when pituitary cells in transgenic NOD mice were made to produce insulin, the cells were not targeted by the immune system even though the pancreatic β cells were. No cellular infiltrate also was shown when the pituitary cells that produced (pro)insulin were taken outside the blood brain barrier and transplanted beneath the renal capsule of NOD mice [21]. Our data and those of Lipes et al suggest that autoimmune destruction of (pro)insulin producing cells in the NOD mouse is specific to the islets.

The blood glucose levels of the diabetic transgenic NOD PEPCK-Ins mice (24.8 ± 1.9 mmol/l) were significantly lower (P = 0.004) than those of the diabetic wild type NOD mice (>33 ± 2.1 mmol/l). This is consistent with some (pro)insulin being released from the liver. These results are in agreement with those of Valera et al in which the majority of their high copy number PEPCK human insulin C57BL/6 mice also became diabetic when injected with streptozotocin despite the constitutive release of human (pro)insulin from the livers of the mice (7). Similarly, the transgenic mice which Valera et al made diabetic by streptozotocin injections also had lower blood glucose levels compared to the diabetic wild type NOD mice suggesting that the constitutive hepatic insulin release lowered the blood glucose levels of the diabetic mice but not to normal. This lack of therapeutic effect could be due to the fact that the PEPCK promoter is induced by glucagon and cyclic AMP. The high glucose levels in the diabetic transgenic mice might have resulted in the inhibition of endogenous glucagon release and thus shutting down (pro)insulin production in the liver.

However it is possible that in some of our transgenic mice, enough (pro)insulin was produced to prevent diabetes. This would explain the significantly lower incidence of diabetes in the transgenic PEPCK-Ins (13%) compared to the wild type (28%) NOD mice (P < 0.001). One possibility is a protective effect of insulin, whether by allowing exhausted beta cells to rest or by altering the makeup of the T cells in the pancreas destroying the β cells there. The first reason was the basis for the large North American trial in pre-diabetic people in the late 90's with small doses of parenteral insulin [22]. The second reason was the basis for the large North American trial in pre-diabetic people in the very late 90's with oral insulin [23].

It is possible that a critical amount of (pro)insulin needs to be produced for an autoimmune effect to be observed. The (pro)insulin content of the transgenic liver cells in normoglycaemic mice was much lower than that of a pancreatic β cell and was also lower than that in the liver of PEPCK-human insulin C57BL/6 transgenic mice produced by Valera et al [7]. (Pro)insulin was not detected immunohistochemically in the liver of our PEPCK-Ins transgenic mice, whereas it was in the liver of Valera's transgenic mice. Attempts by us to increase production of (pro)insulin by producing homozygous mice failed, with all mice being stillborn (Figure 1C). This might be because of transient upregulation of the PEPCK gene at birth [24], resulting in increased production of (pro)insulin that caused hypoglycaemia. Alternatively, it could be due to a transgene integration effect where the transgene disrupted a vital gene; with a double copy deletion being lethal.

Another possible explanation for the lack of an autoimmune effect is that presentation of insulin peptides by hepatocytes might be limited by the ability of the liver cells to cleave proinsulin. The enzymes responsible for this in the β cell, prohormone convertases (PC) 1/3 and PC2, are absent from normal liver cells, but the endopeptidase furin is present [25]. Cleavage of proinsulin by furin however would require genetic modification of the peptide [8].

In summary, we have shown that transgenic NOD mice that produce (pro)insulin in their liver do not develop cellular infiltration of their liver when autoimmune destruction of pancreatic β cells occur. Furthermore the expression of (pro)insulin in hepatocytes is insufficient to prevent development of diabetes in NOD mice. These results offer hope that eventually liver cells, or a subpopulation of them, may be of value as a therapy for type 1 diabetes.
